# A Sensitive SPE-LC-MS/MS Method for Determination of Selected Veterinary Drugs and Other Organic Contaminants in Human Urine: Development, Validation, and Application Study

**DOI:** 10.3390/ijms26189025

**Published:** 2025-09-16

**Authors:** Wojciech Rodzaj, Małgorzata Wacławik, Joanna Jurewicz, Bartosz Wielgomas

**Affiliations:** 1 Department of Toxicology, Faculty of Pharmacy, Medical University of Gdańsk, 107 Hallera Street, 80-416 Gdańsk, Poland; wojciech.rodzaj@gumed.edu.pl (W.R.); malgorzata.waclawik@gumed.edu.pl (M.W.); 2 Department of Chemical Safety, Nofer Institute of Occupational Medicine, 8 Teresy Street, 91-348 Łódź, Poland; joanna.jurewicz@imp.lodz.pl

**Keywords:** pesticides, flame retardants, bisphenol S, human biomonitoring, urine, liquid chromatography–mass spectrometry

## Abstract

The complexity of human exposure to surrounding chemicals warrants developing analytical methods that are capable of the simultaneous quantitation of many diverse environmental pollutants and their biomarkers for the needs of human biomonitoring (HBM). Examples include pesticides used in veterinary medicine, such as fipronil (FIP), imidacloprid and pyrethroids, as well as other chemicals, like bisphenols and flame retardants. The goal of this paper was to develop and validate a liquid chromatography-tandem mass spectrometry method for the quantification of selected organic contaminants in human urine. The method was then applied to real samples and used to assess the potential of a new FIP biomarker, fipronil-hydroxy (FIP-OH), for HBM. As a sample preparation protocol, enzymatic deconjugation followed by solid phase extraction were used. The method was successfully developed and validated for 16 organic pollutants and/or their metabolites, with lower limits of quantitation ranging from 0.5 to 2000 pg/mL. FIP-OH could not be included in the method, possibly due to its chemical instability. In an application study among pet owners, the detection rate for FIP was 71% (median: 3.32 pg/mL); several other chemicals were also commonly detected. The results of validation and application experiments confirm that the method can be used in HBM studies to quantify organic pollutants in urine.

## 1. Introduction

Humans are exposed to many chemicals on a daily basis. The variety of chemicals and sources thereof in the surrounding environment made the single, isolated exposures an exception rather than the rule. As exposure to chemicals may cause negative health effects, monitoring the magnitude is needed in order to determine the associated risk and take action, if necessary [1]. Human biomonitoring (HBM), based on the collection of biological samples from subjects and the determination of compounds of interest, is a crucial tool for exposure assessment [2]. It allows us to track exposure from all sources and routes [3] and its results can be directly translated into an internal dose, which may exert systemic effects in the human body [4]. The chemicals that are highly prioritized for exposure assessment due to their possible toxicity and widespread exposure include pesticides, bisphenols, flame retardants, and per-/polyfluoroalkyl substances (PFASs) [5,6].

Most commonly, pesticides are used to protect crops [7]. In some cases, however, their usage may extend to other areas, such as residential applications and veterinary medicine. That was the case with several flagship insecticides, including fipronil (FIP), a phenylpyrazole [8], imidacloprid (IMI), a neonicotinoid [9], and permethrin, a pyrethroid [10,11]. Other insecticides, such as flumethrin, were designed for animal treatment from the beginning [12].

FIP acts via blocking γ-aminobutyric acid-related neural transmission, leading to the paralysis and death of arthropods such as locusts, cockroaches, ticks, and fleas [13]. After its registration in many countries for crop protection, residential use, and veterinary applications in the 1990s, FIP was banned in the European Union (EU) from agriculture use in 2016 due to its toxicity towards bees [14]. Its approval for biocide use in the EU expired in 2023 [15], making the ectoparasiticidal treatment of household pets the only approved application in that region. In mammals, FIP may cause acute neurotoxic effects, whereas chronic exposure may lead to thyroid, developmental and reproductive toxicity [16].

In humans, no exposure biomarker to FIP has been established yet. In epidemiological studies, FIP itself and its active metabolite, fipronil-sulfone (FIP-sulfone), are mainly detected [17]. These compounds, however, can also be found in the environment [13], which poses a risk of external contamination of the biological samples during collection. Additionally, due to their lipophilicity, both FIP and FIP-sulfone are poorly excreted with urine [18], a matrix often used in HBM [19]. To overcome these problems, fipronil-hydroxy (FIP-hydroxy), hitherto detected only in rats, was proposed as a promising urinary biomarker of FIP exposure in humans [20]. Other FIP derivatives, that are more relevant to environmental research, include fipronil-detrifluoromethylsulfinyl (FIP-dtfms), fipronil-amide (FIP-amide), fipronil-desulfinyl (FIP-desulfinyl), and fipronil-sulfide (FIP-sulfide). FIP and its metabolites/degradation products are collectively known as fiproles (FIPs) [15].

IMI kills arthropods by binding to nicotinic receptors causing the overexcitation of the arthropods’ nervous system [21]. Introduced to the market shortly before FIP, it met a similar fate—worldwide commercial success [22] followed by restrictions of use due to environmental concerns [23]. In the EU, it is still widely used to prevent and treat ectoparasite infestations in household pets [15]. Suspected adverse effects of IMI exposure in humans involve neurotoxicity, hepatotoxicity and reproductive toxicity [24]. Biomarkers of human exposure to IMI include the parent compound and its 5-hydroxy derivative (IMI-OH) [25].

Insecticidal action of pyrethroids is based on the alteration of voltage-sensitive sodium channel kinetics in the arthropod nervous system [26]. Permethrin and flumethrin, both pyrethroid insecticides, are popular ectoparasiticides used in veterinary products for household pets [15]. In humans, pyrethroid poisoning is associated with neurotoxic effects [26]. While many studies track permethrin exposure by measuring urinary 3-phenoxybenzoic acid levels [27], this chemical can be formed in the environment [28], whereas no such reports exist for 4′-hydroxy-3-phenoxybenzoic acid (4OH3PBA), another metabolite of permethrin [29]. To the authors’ knowledge, no HBM study using biomarkers specific to flumethrin has been published yet. Animal studies indicate that 3-(2-chloro-2-(4-chlorophenyl)vinyl)-2,2-dimethylcyclopropanecarboxylic acid (CPhCA) may be used for that purpose, whereas 4-fluoro-3-phenoxybenzoic acid (4F3PBA) is a less specific alternative [12].

In contrast to the insecticides described above, the use of fungicides is heavily focused on crop protection [30]. Important examples include imazalil, boscalid, and tebuconazole [31]. While their mechanisms of action vary, all three are excreted with urine as conjugates of hydroxy metabolites produced during phase I of metabolism: imazalil O-dealkenylation leads to the formation of imazalil-despropenyl (IMZ-OH) [32], whereas aromatic oxidation of boscalid and tebuconazole yields boscalid-5-hydroxy (BOS-OH) [33] and tebuconazole-*tert*-butylhydroxy (TEB-OH) [34], respectively. In non-target organisms, these fungicides may induce endocrine disruption [35,36] and neurotoxicity [37].

While not a pesticide itself, DEET (*N*,*N*-diethyl-*meta*-toluamide) is often discussed alongside these chemicals. Instead of killing insects, DEET is used to repel them. Since DEET is often applied directly onto human skin, internal exposure can be expected [38]. In general, DEET is considered safe, but may induce neurotoxicity, especially if used in combination with insecticides [39].

Bisphenol S (BPS) is a substitute to bisphenol A, commonly used in the production of polycarbonate materials and epoxy resins. Studies show, however, that BPS exhibits similar endocrine activity to the chemical it is supposed to replace [40,41,42]. In humans, BPS is rapidly excreted with urine, mostly in its conjugated form [43].

Organophosphate flame retardants (OPFRs) are commonly used to reduce flammability of various materials [44]. Aryl OPFRs, such as triphenyl phosphate and 2-ethylhexyl diphenyl phosphate, constitute an important group of these chemicals [45]. These and some other OPFRs are known to be metabolized to diphenyl phosphate (DPhP), which is commonly detected in many populations [46]. Investigations on aryl OPFRs show that these chemicals may exert endocrine- and metabolic-disrupting effects [47].

Perfluorooctanoic acid (PFOA) is a widely known PFAS [48]. Due to its persistence, it can still be expected to be found in humans, despite its use being strongly restricted over the years [6]. PFOA exhibits carcinogenic, endocrine-disrupting, immunotoxic, and hepatotoxic properties [48].

Many chemicals, despite their different structures, may exhibit similar negative health effects [1,49,50]. Synergism between these substances may cause a stronger effect than a single chemical [51], so an effort to monitor as many potentially harmful chemicals as possible is warranted. In order to achieve this goal efficiently, wide-scope analytical methods are needed. Therefore, the main goal of this study was to develop and validate a liquid chromatography-tandem mass spectrometry (LC-MS/MS) method for the determination of a diverse group of organic contaminants in human urine. Additionally, the developed protocol was used to verify the potential of FIP-hydroxy as a biomarker of human exposure. Finally, the method was used to quantify the compounds of interest in real samples in order to examine its applicability.

## 2. Results and Discussion

### 2.1. Hydroxy-Fipronil Investigation

**Figure 1:**
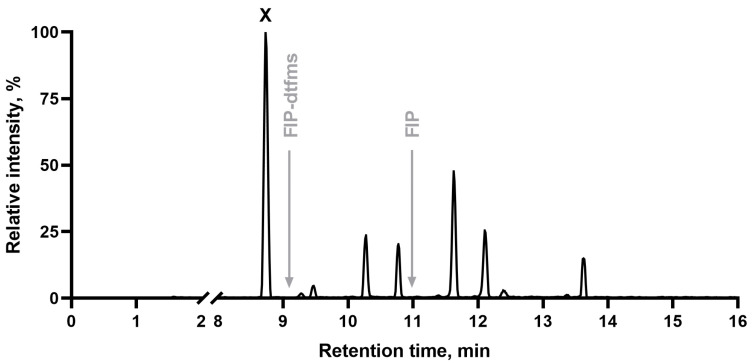
Total ion chromatogram of the FIP-hydroxy standard in negative ionization. The peak that was subjected to further investigation is marked by an “X”. Retention times for FIP-dtfms and FIP are indicated by gray arrows.

The total ion chromatogram in negative mode of the FIP-hydroxy standard donated by prof. Bruce Hammock from UC Davis is shown below (Figure 1). Several peaks were observed instead of a single one. Since FIP-hydroxy is a hydroxylated derivative of FIP-dtfms, its retention time should be shorter than both FIP-dtfms and FIP. Only one peak, marked with an “X” on Figure 1, matched that criterion.

**Figure 2:**
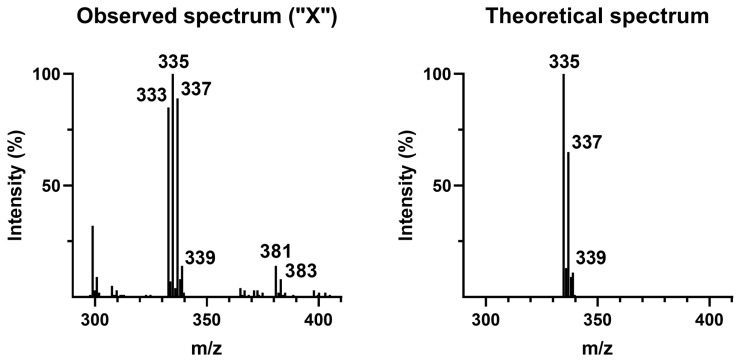
Spectrum of peak “X” (**left**) and theoretical spectrum expected for FIP-hydroxy (**right**).

For the “X” peak, a mass spectrum in negative mode was obtained (Figure 2, left). As a reference, a mass spectrum of [M-H]^−^ precursor for a molecular formula corresponding to FIP-hydroxy (C_11_H_5_Cl_2_F_3_N_4_O) was generated using Molecular Weight Calculator, version 6.50 (Figure 2, right). Note that an intense signal at *m*/*z* 333 is present in the observed spectrum, but not in the predicted one. The intensity ratios of *m*/*z* 335, 337, and 339 are different as well. The mismatch might have been caused by coelution with another compound or partial in-source oxidation of the hydroxy group, possibly to a ketone derivative [52,53]. At the same retention time, *m*/*z* 381 and 383 in a ratio corresponding to the presence of two chlorine atoms were also observed (Figure 2, left). These *m*/*z* match the values expected for a formate adduct of FIP-hydroxy and were used as a proof of FIP-hydroxy formation in rats in the research of Vasylieva et al., 2017 [20]. Since the mobile phase used in the present study contained formate ions (see Section 3.2), such adducts could be formed here as well. However, the discrepancy between the *m*/*z* values observed for pseudomolecular ions and potential adducts makes drawing certain conclusions difficult. In the paper proposing FIP-hydroxy for use in HBM, some concerns were raised about the purity of the standard [20]. However, the unexpected signals observed here may also result from the chemical instability of the compound. To rule out the possibility of FIP-hydroxy degradation during storage, a new FIP-hydroxy standard was ordered from one of the largest manufacturers of analytical standards in the industry. However, after repeated attempts, the company failed to synthesize the product.

Finally, the MS/MS conditions for *m*/*z* 335 in negative mode (Figure 2, left) were optimized and 42 samples with quantifiable FIP(s) levels were screened for transitions obtained. A transition in positive mode, used by Vasylieva et al., 2017 [20], was included as well. The samples were prepared according to the final protocol (Section 3.4). None of the samples tested were positive for FIP-hydroxy. Consequently, FIP-hydroxy was not included in the method.

Our failure to establish FIP-hydroxy as a urinary biomarker of FIP exposure in humans may stem from several reasons. Firstly, FIP-hydroxy has only been found in rats so far [18,20], and both quantitative and qualitative differences in metabolism between rats and humans are widely reported in the literature [54,55,56,57]. Although a comparative study of human and rat liver microsomes stresses the qualitative similarity between FIP metabolism in both species [58], only one metabolite, FIP-sulfone, was taken into account in that study. Secondly, the insufficient stability of the compound itself might have led to a situation where FIP-hydroxy, despite being produced in the human body and excreted with urine, was still undetectable due to degradation during sample storage and/or preparation. Finally, assay sensitivity may have affected the results. It appears that the trifluoromethylsulfinyl moiety of the FIP molecule plays an important role in ionization efficiency in the electrospray ion source. For instance, at the same molar concentration, FIP generates a signal that is approximately 30 times higher than FIP-dtfms, which is deprived of that functional group. A similar pattern can be expected for FIP-hydroxy, making it undetectable despite being excreted with urine. Since several other urinary metabolites have been reported in laboratory animals [18,59], searching for a different urinary biomarker might yield better results in the future.

### 2.2. Method Development

The final protocol is described in Section 3.4. Flow-through solid phase extraction (SPE) was chosen as a sample preparation technique because it is an exhaustive extraction method and, in consequence, it provides maximum sensitivity [60]. Some of the steps of sample preparation were adapted from other methods developed in the laboratory. The deconjugation procedure was already used in the research of Klimowska and Wielgomas, 2018 [61], whereas ethyl acetate was demonstrated to be an optimal elution solvent in the research of Klimowska et al., 2023 [62]. FIP-hydroxy was not included in method development due to the reasons described in the previous section.

#### 2.2.1. Filtration Loss Experiment

The goal of a filtration process before chromatographic analysis is to remove particles that are present in the extract which could clog the tubing and/or the column of the LC system. It is typically the final step of sample preparation [63]. However, the wrong choice of filter material and/or filtration conditions may cause substantial analyte loss [63,64]. The process of binding analytes to the filter material may involve van der Waals forces, hydrophobic interactions, hydrogen bonding, electrostatic interactions, or other phenomena. The organic content of the solution is one of the key parameters affecting this process [63]. Since the filtration step was present in all optimization experiments, the investigation on the effect of the solvent composition on analyte loss during filtration was conducted first. Nylon was used as the filter membrane material.

**Figure 3:**
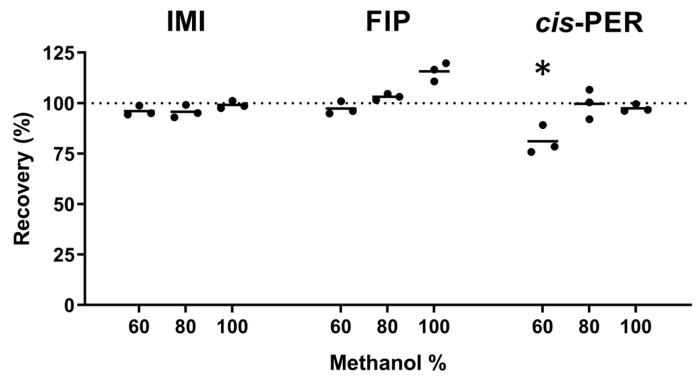
Analyte loss due to filtration. The solid lines represent the mean values. A dotted line at 100% recovery was added for reference. An asterisk denotes a statistically significant difference (*p* < 0.05).

The results of the optimization of solvent composition before filtration are shown below (Figure 3). IMI, FIP and *cis*-permethrin (*cis*-PER) were used as model compounds for the experiments. Although *cis*-PER was not among the analytes quantitated in urine, it was included in this experiment to take into account compounds that are more lipophilic than FIP. As shown in Figure 3, the solvent composition was not an important factor for the recovery of IMI and FIP (average recovery within 96–116%). However, a statistically significant loss was observed for *cis*-PER at 60% methanol (average recovery of 81%; *t*-test, *p* = 0.0168) compared to the unfiltered reference. No statistically significant loss was observed in the case of 80 or 100% methanol. The loss of *cis*-PER at the lowest methanol content was not unexpected, as the lipophilic analytes are generally more strongly retained by filter membranes if the water content of the extract is high [63]. At the same time, lower methanol content in the final extract would be beneficial to the peak shape of early-eluting analytes [65]. As a compromise, 80% methanol was used to redissolve evaporated extracts before filtration.

#### 2.2.2. Extraction Cartridge Selection

**Figure 4:**
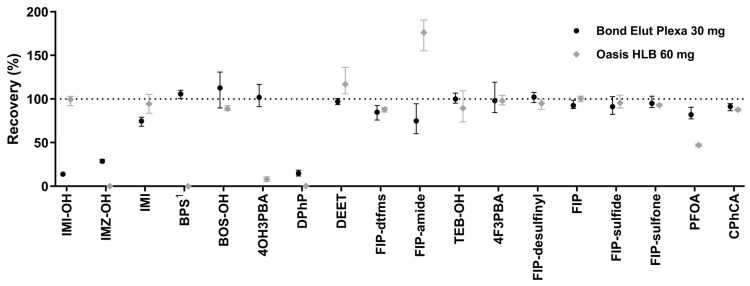
Recovery comparison for compounds of interest obtained using Bond Elut Plexa 30 mg and Oasis HLB 60 mg. The data points represent the mean, whereas the whiskers show the range. A dotted line at 100% efficiency was added for reference. ^1^ Due to the presence of unlabeled BPS in the matrix, a deuterated analog was used to assess the recovery.

Careful consideration of the stationary phase used during the SPE procedure is necessary for efficient sample preparation [66]. To select an optimal stationary phase for SPE, Bond Elut Plexa 30 mg and Oasis HLB 60 mg extraction cartridges were compared. The results are shown in Figure 4. The recovery for most analytes was close to 100% regardless of the cartridge used. For some polar analytes, however, sharp differences between the sorbents were observed. While better recovery was observed for IMI-OH using Oasis HLB compared to Bond Elut Plexa (99 and 14%, respectively), the former performed much worse in the case of some acidic species, namely BPS, 4OH3PBA, DPhP, and PFOA (Figure 4). For instance, the average recovery of 4OH3PBA extraction was 8 and 102% for Oasis HLB and Bond Elut Plexa, respectively. In the case of FIP-amide, a surprisingly high result was obtained using Oasis HLB (176%). Since Bond Elut Plexa performed better than Oasis HLB in several cases, the former was chosen for the final protocol.

It is not clear why Oasis HLB performed so much worse for several acidic species (Figure 4), especially since its bed mass was two times higher than in Bond Elut Plexa (60 vs. 30 mg, respectively). The discrepancies between tested cartridges might stem from different chemistries of sorbent material. While the Oasis HLB sorbent is described by Waters as a copolymer of divinylbenzene and N-vinylpyrrolidone [66], Agilent describes Bond Elut Plexa as “divinylbenzene-based” [67]. However, further research would be necessary to provide a definitive answer to this question. Finally, an unexpectedly high signal of FIP-amide in the case of Oasis HLB may result from signal enhancement due to the presence of interferences in the blank urine used for the experiment.

#### 2.2.3. Washing Step Optimization

After selecting the SPE cartridge, the sorbent wash conditions for Bond Elut Plexa 30 mg were optimized in order to wash away as many interferences as possible without losing the compounds of interest. The formic acid concentration (1%, *v*/*v*) was kept constant, while methanol percentages ranging from 0 to 25% were tested. Figure S1 depicts the results. In most cases, the analyte signal was unaffected by the methanol percentages tested. However, a sharp decrease in recovery was observed for IMZ-OH when the methanol content was increased from 15 to 20% (Figure S1). The change was statistically significant (*t*-test, *p* = 0.0004). Additionally, a slow but consistent signal increase for IMI and BPS was observed with increasing methanol content in the wash solution (Figure S1). Since the decreasing IMZ-OH signal turned out to be the limiting factor for sample cleanup, the methanol percentage for the washing step was set at 15%.

The rapid decrease in observed IMZ-OH signal with increasing methanol content was not surprising. Its parent compound, imazalil, is a weak base [68], and similar behavior was expected for IMZ-OH. The presence of formic acid in the wash solution led to IMZ-OH protonation, making it susceptible to elution with an aqueous–organic mixture. The increase in IMI and BPS signal with increasing methanol percentage is probably the result of decreasing signal suppression, since fewer interferents could be found in the final extract of the samples treated with a stronger wash solution.

### 2.3. Method Validation

Following optimization, the method was validated. The goal of method validation is to ensure that the assay performance is sufficient and the results it provides are reliable. Here, the validation process was conducted based on the ICH M10 guideline on bioanalytical method validation and study sample analysis [69], with the exception of the matrix effect investigation, which was carried out following the procedure described in EMA, 2011 [70]. Since the results for IMZ-OH and PFOA failed to meet the validation criteria, these analytes were omitted.

#### 2.3.1. Selectivity

The selectivity of the analytical method was investigated by injecting blank urine samples and evaluating the analyte signal. No significant amounts of any of the analytes and/or internal standards (ISs) were found. Therefore, the selectivity of the method was assessed as satisfactory.

#### 2.3.2. Internal Standard Selection and Matrix Effect

Mass spectrometric methods are prone to a matrix effect, which is a change in analyte response due to interferences present in the matrix. Since it may reduce the accuracy, precision, and robustness of the method [71], its presence and magnitude were carefully evaluated. Absolute matrix effects (matrix factors) for the compounds of interest are provided in Table 1. Specific gravity (SG) for all urine lots is also shown. In all cases, signal suppression was observed compared to pure solvent. Additionally, an increasing SG value was often associated with a stronger matrix effect. These observations were not unexpected as samples with higher SG contain more matrix components which contribute to matrix effects [71]. According to Ferrer Amate et al., 2010 [72], the uncorrected matrix effect within 80 and 120% corresponds to a soft matrix effect, whereas matrix effects between 50 and 79% and between 121 and 150% can be considered to be medium signal suppression and enhancement, respectively; finally, values below 49 and above 151% indicate a strong matrix effect. Based on this description and the average matrix factors (Table 1), nearly all analytes were subjected to strong signal suppression. Medium signal suppression was observed for DPhP and TEB-OH only.

This observation can be attributed to the fact that urine samples were concentrated one hundred times during sample preparation with relatively little cleanup (wash solvent used during SPE contained only 15% methanol), resulting in high amounts of interferents being injected into the LC-MS/MS system. Even under the assumption that the unconcentrated sample would cause no matrix effect, the gain in sensitivity resulting from sample preconcentration was still higher than the observed signal suppression, although the signal increase varied greatly. For instance, the average signal of TEB-OH in the matrix was 48% lower than in pure solvent (Table 1). However, taking into account the sample preconcentration, its signal was still approximately 50 times higher than the result that could be expected in a sample not subjected to preconcentration. On the other hand, a 98% average decrease in BOS-OH signal due to a matrix effect in the preconcentrated sample lead to an only twofold increase in the analytical response overall, making the signal gain almost insignificant. These two extreme examples show how diverse results can be obtained using high sample preconcentration as a strategy for improving the sensitivity of an LC-MS/MS method.

The IS-corrected matrix factors for optimal analyte–internal standard pairs are provided in Table 2. The coefficients of variation (CVs) of IS-corrected matrix factors, which reflect the relative matrix effect [73], are given in the last column of the Table 2. No clear link between the average value and urine lot SG could be found. Unsurprisingly, analytes with an isotopically labeled analog used as an internal standard exhibited low variability of the IS-corrected matrix factor. For instance, the CVs of IS-corrected matrix factors for IMI, FIP-dtfms and FIP were 6, 3, and 2%, respectively. BPS is a notable exception, with a CV of an IS-corrected matrix factor of 12%, despite the use of a deuterated analog as an internal standard (Table 2). Interestingly, a similar response variability (13%) was observed in the work of Gys et al., 2020 [74], even though the ^13^C-labeled internal standard was used in that work. For other analytes, CVs ranging from 9 (FIP-sulfone) to 41% (BOS-OH) were observed. In order to mitigate the variability associated with a relative matrix effect, matrix-matched internal calibration was used for quantitative analysis of all analytes [62,75,76].

#### 2.3.3. Linearity and Lower Limit of Quantification

The lower limit of quantification (LLOQ) is “the lowest amount (concentration) of an analyte in a sample that can be quantitatively determined using a method with predefined precision and accuracy” [69]. Linearity is the assumption that the signal–concentration relationship for a given analyte forms a straight line [77]. The results of experiments on assay linearity and sensitivity are shown in Table 3. The LLOQs ranged from 0.5 (FIP-sulfone) to 2000 pg/mL (4OH3PBA). The linear range spanned two or more orders of magnitude for all analytes, except for DPhP (100–5000 pg/mL). The dilution integrity experiment allowed us to extend this range for some analytes even further (see Section 2.3.6.). The calibration curve fit was determined using the coefficient of determination (R^2^) and its value did not fall below 0.9900 for any analyte (Table 3).

The sensitivity of the method developed here can be compared to those achieved recently in other laboratories. For IMI biomarkers, namely IMI itself and IMI-OH, our results were similar to the limits of quantification (LOQs) observed in Wrobel et al., 2023 (190 and 1000 pg/mL, respectively) [78], but much higher than the limits of detection (LODs) reported in Baker et al., 2019 (30 and 50 pg/mL, respectively) [79]. In our method, a significant loss of IMI-OH occurred during the SPE procedure (Figure 4), strongly affecting the observed LLOQ. Nevertheless, the sensitivity for both analytes seems sufficient to monitor human exposure to IMI after ectoparasiticide application on household pets as observed in the work of Wrobel et al., 2024 [80]. The LLOQ for BPS (Table 3) was close to the values reported by other authors, such as 40 [74] and 250 pg/mL [81]. In contrast, the sensitivity of the hereby presented method for BOS-OH (1000 pg/mL) was poor compared to LODs reported by Rodríguez-Zamora et al., 2024 (50 pg/mL) [75] or Norén et al., 2024 (20 pg/mL) [82]. This discrepancy can be attributed to the very strong signal suppression of BOS-OH, as evident in the results presented in Table 1. For DPhP, the assay performed quite well, although the linear range was narrow (100–5000 pg/mL), and urinary concentrations above the upper limit of quantification (ULOQ) reported here are not out of the ordinary [46]. However, the achieved LLOQ was satisfactory—for comparison, an LOD equal to 20 pg/mL was reported in the work of Norén et al., 2024 [82], whereas in the work of Van den Eede et al., 2013, the LOQ was 300 pg/mL [83]. The 10 pg/mL LLOQ for DEET (Table 3) is considerably lower than the values reported in the recent literature, ranging from 80 [84] to 210 pg/mL [85]. The LLOQ established for TEB-OH (100 pg/mL) lies between the LOD reported by Norén et al., 2024 (16 pg/mL) [82] and the LOQ from the work of Šulc et al., 2022 (270 pg/mL) [86]. For 4F3PBA, other authors usually reported better sensitivity, such as 30 [87] or 25 pg/mL [88]. No data to compare could be found for other pyrethroid metabolites (4OH3PBA and CPhCA). The LLOQs for FIP-desulfinyl, FIP, FIP-sulfide and FIP-sulfone were 5, 1, 1, and 0.5 pg/mL, respectively (Table 3). Quantitation limits reported in the literature for these compounds ranged from 0.1 [76] to 2.5 pg/mL [89]. For FIP-dtfms and FIP-amide, no data from the literature was available for comparison.

Two important things should be mentioned here. Firstly, while the sensitivity of the developed method was usually at a similar level with the ones developed by other authors, the one presented here requires a much higher sample volume (5 mL). In the papers mentioned above, the sample volume ranged from 0.2 to 1 mL, with the exception of the work of Le Grand et al., 2012 [88], where 5 mL of urine was needed. Secondly, the assay sensitivity can be established in many different ways [90], which makes the comparisons difficult. The method used here, based on the accuracy at the LLOQ level, as recommended by the European Medicines Agency (EMA) [69], can be considered conservative compared to other methods, such as the ones based on the analyte signal-to-noise ratio, which is heavily influenced by the data collection process within the software [91].

#### 2.3.4. Accuracy and Precision

Accuracy can be defined as the closeness of the measurement to the nominal value, whereas precision is the degree of agreement between a series of measurements [69]. The results of accuracy and precision experiments on quality control (QC) urine samples are shown in Table 4. For the vast majority of the analytes, the criteria set by the EMA guidelines [69] were fulfilled for QC samples at both low (LQC) and high (HQC) concentration. The only exception was the LQC inter-run precision for IMI-OH and DPhP (20 and 16%, respectively). The use of isotopically labeled analogs of these compounds as internal standards would certainly improve this parameter; it is planned to include them in future studies.

#### 2.3.5. Carry-Over

Carry-over is a change in measured analyte concentration due to its residues from the previous injection still being present in the system. The carry-over experiment revealed no significant transfer of any analyte and/or internal standard residues from one injection to another.

#### 2.3.6. Dilution Integrity

This experiment was performed in order to determine whether sample dilution affected the accuracy and precision of the results at the levels exceeding the highest level of calibration curve. The results are shown in Table S1. The eightfold dilution integrity was confirmed for IMI, BPS, FIP-dtfms, FIP-desulfinyl, FIP, FIP-sulfide, FIP-sulfone, and CPhCA.

#### 2.3.7. Stability

In biomonitoring studies, the samples may undergo many processes before analysis, such as shipping, preparation, or short- and long-term storage [92]. Therefore, analyte stability, understood as a lack of analyte degradation in a given matrix during defined storage conditions [69], is another important validation parameter [93]. The stability studies included the 24 h autosampler stability of processed samples at room temperature and the long-term stability of unprocessed QC samples stored for 30 days and 12 months at −20 °C. The results are presented in Table 5. All analytes were stable in processed samples stored in the autosampler at both QC levels. The long-term stability study, however, revealed significant losses of BOS-OH and 4OH3PBA after 12 months at both LQC (accuracy of 46 and 77%, respectively) and HQC levels (46 and 58%, respectively). Notably, all analytes were stable in a solvent stored for the same period of time.

To the authors’ knowledge, this is the first study to investigate 1-year stability of these compounds in urine. Although it probably does not mimic the real-life scenario perfectly (in animal studies, both BOS-OH [33] and 4OH3PBA [29] are mostly excreted as conjugates), the results presented here raise concerns about their use for human biomonitoring. Spiking the urine samples before storage with stabilizers, such as ascorbic acid or butylated hydroxytoluene [94], may also be considered to prevent the degradation of these analytes. Further research is necessary to determine whether such an approach would be efficient for BOS-OH and 4OH3PBA.

### 2.4. Method Application

The developed method was applied to 28 urine samples collected during a longitudinal study on pet owners who recently applied an ectoparasiticide on their pet. The medication contained either fipronil, permethrin, or both insecticides. The summary statistics, both uncorrected and adjusted by SG, are presented in Table 6. For IMI-OH, IMI, FIP-dtfms, FIP-amide, 4F3PBA, FIP-desulfinyl, FIP-sulfide, and CPhCA, the detection rates were negligible (≤4%). 4OH3PBA was not commonly detected either (18% of the samples). A moderate detection rate was observed for BOS-OH and FIP-sulfone (50 and 39%, respectively). The geometric mean concentration for BOS-OH was 1238 and 1454 pg/mL (unadjusted and SG-adjusted result, respectively; all concentrations will be given in that order). Finally, BPS, DPhP, DEET, TEB-OH, and FIP were frequently detected (89, 93, 68, 89, and 71% of the samples). The highest median concentration was observed for DPhP (1712 and 1656 pg/mL), whereas the lowest was reported for FIP (3.32 and 3.39 pg/mL).

A near lack of samples that were positive for IMI-OH and IMI was not surprising, as the samples were collected after the EU ban of IMI use in plant protection products [23]. In a German study where samples were collected before the ban was imposed [78], the detection rates for IMI-OH and IMI were also fairly low (21 and 36%, respectively), even though some samples were collected following the application of an IMI-based animal care product on a pet. In contrast, BPS was frequently detected in the present study, with the uncorrected concentration median as high as 663 pg/mL (Table 6). Much lower concentrations were observed in samples collected from Polish children in 2014–2015 (median below the LOQ of 250 pg/mL). This discrepancy can be explained at least partially by the different period of sample collection. In the study on Polish children, the samples were collected before the EU ban of bisphenol A for use in thermal paper, which was imposed in 2020. It was followed by increased use of other bisphenols, such as BPS [95]. Since the samples for the present study were collected in 2021–2022, higher BPS levels could be expected. Indeed, the exposure to BPS has been reported to increase over the past several years in Sweden [82] and Denmark [96]. Limited data exist for urinary levels of BOS-OH in humans; a SG-corrected geometric mean (GM) equal to 330 pg/mL was reported in farmworkers in Costa Rica [75], whereas the 50th percentile in Swedish adolescents was 160 pg/mL (uncorrected concentration) [82]. In our study, a SG-corrected GM of 1454 pg/mL was observed, suggesting much higher exposure. Relatively high urinary levels were also observed for DPhP (uncorrected median: 1712 pg/mL). In comparison, the median concentration in a nationwide US study was 820 pg/mL [97], whereas in Swedish adolescents it was equal to 930 pg/mL [82]. The value observed here, however, was still lower than the median reported in a study on American firefighters (2900 pg/mL) [98]. The DEET detection rate was also higher than in other studies; for instance, a method application study on 75 anonymous adult volunteers reported 12% detection frequency [38]. This difference may stem from the higher LOD than the LLOQ reported here (100 and 10 pg/mL, respectively). TEB-OH prevalence and concentrations observed in our study were slightly lower than in an adult population of the Czech Republic (detection rate ≥ 95%, SG-corrected median of 470 and 440 pg/mL for winter and summer seasons, respectively) [86]. Finally, the relatively high detection frequency and concentrations of FIP and FIP-sulfone observed in our study probably result from the ectoparasiticide application prior to sample collection. This observation suggests that FIP and FIP-sulfone can be used as biomarkers of human exposure to FIP in humans. To compare, FIP-sulfone was detected in less than 4% of Czech adults [86] and in only 10% of a general population in China [89]. A similar detection rate of FIP and FIP-sulfone (48 and 40%, respectively) was observed only in a longitudinal study on urinary excretion variability conducted in Luxembourg/France [99]. Five out of sixteen volunteers who participated in that study reported having a pet treated with ectoparasiticide, and that is probably why the detection rate was comparable to the results presented herein. In line with other biomonitoring studies on fiproles [17], FIP-amide, FIP-desulfinyl, and FIP-sulfide were not detected.

It should be noted that due to the small sample size, the results presented here may not be representative of the general population. Additionally, due to the study intervention mentioned earlier, the levels of fipronil and pyrethroid biomarkers observed in this sample are likely to be elevated compared to others.

## 3. Materials and Methods

### 3.1. Chemicals and Materials

Analytical standards used in this work (both analytes and internal standards) are presented in Table S2. β-Glucuronidase from *Helix pomatia* type HP-2 (low sulfatase activity, catalog number G7017), glacial acetic acid, and formic acid pro analysis were purchased from Sigma-Aldrich (Burlington, MA, USA). Isopropanol, acetonitrile, methanol, water and formic acid (all LC-MS grade) were obtained from Supelco (Bellefonte, PA, USA). Ethyl acetate GC-MS grade and Extran MA 01 laboratory detergent were obtained from that supplier as well. Anhydrous sodium acetate was purchased from POCH (Gliwice, Poland). Technical nitrogen and argon 6.0 were acquired from Oxygen (Gdańsk, Poland) and Air Products (Allentown, PA, USA), respectively.

Bond Elut Plexa (30 mg) and Oasis HLB 3 cc (60 mg) cartridges were purchased from Agilent Technologies (Santa Clara, CA, USA) and Waters (Drinagh, Ireland), respectively. Centrifugal filters (0.2 µm pore size, modified nylon), rimless test tubes, and disposable transfer pipettes were obtained from VWR (Leuven, Belgium). Screw-top borosilicate glass tubes were provided by Corning (Corning, NY, USA), whereas phenolic screw thread caps (15-415) were obtained from Sun-Sri (Rockwood, TN, USA). Sarstedt (Nümbrecht, Germany) was the supplier of all pipette tips. Chromatography vials, along with caps and microinserts, were purchased from La-Pha-Pack (Langerwehe, Germany).

### 3.2. Instrumental Analysis

The chromatographic separation was performed using a Varian system (Varian, Walnut Creek, CA, USA) consisting of two 212-LC dual piston pumps, a ProStar 420 autosampler, and a high pressure mixer. The heated column compartment from Dionex (Sunnyvale, CA, USA) was used. ACE Excel 3 SuperC18, 75 × 3.0 mm (particle size 3 µm) from Advanced Chromatography Technologies (Aberdeen, Scotland) was used as an analytical column. Mobile phase A consisted of 0.5 mM of ammonium formate buffer at a pH of 3 in water:methanol in a 9:1 ratio (*v*/*v*), whereas phase B was the same buffer in pure methanol. The gradient program, along with the list of other chromatographic parameters, is shown in Table S3.

A 320-MS triple quadrupole mass spectrometer with an electrospray interface (Varian) was used for analyte detection. A detailed list of MS parameters is shown in Table S4.

In Table 7, compound-specific parameters for instrumental analysis are shown. MS Workstation software, version 6.9.3 (Varian) was used for system control, data collection, and analysis.

### 3.3. Fipronil-Hydroxy

As mentioned in the Introduction, FIP-hydroxy is a promising candidate for a urinary biomarker of human FIP exposure. However, it has only been detected in rats so far [20]. After an analytical standard was kindly provided by prof. Bruce Hammock from UC Davis, a series of experiments were conducted in order to investigate the FIP-hydroxy potential for human biomonitoring. Molecular Weight Calculator software, version 6.50 (Matthew Monroe, Richland, WA, USA), was used to generate a theoretical mass spectrum of the FIP-hydroxy parent ion. Since the precursor *m*/*z* observed during the infusion of the FIP-hydroxy standard into the mass spectrometer did not match the expected values, additional LC-MS(/MS) analyses of FIP-hydroxy were performed. LC conditions were the same as in Table S3. Scans were run in both positive and negative mode (*m*/*z* range 100–920) at 70 V of capillary voltage. Several peaks were observed in negative mode and the MS/MS parameters were determined separately for all peaks selected for further analysis.

### 3.4. Final Protocol of Sample Preparation

All reusable glassware used during method development and sample preparation was thoroughly cleaned using ultrasonic bath and laboratory detergent, then rinsed with methanol, and baked in a muffle furnace at 350 °C for 4 h; single-use glassware was baked in the furnace as well.

In the final method, 5 mL aliquots of urine samples in glass screw cap tubes were spiked with an IS mixture and incubated overnight at 37 °C with 1250 µL of β-glucuronidase type HP-2 from *Helix pomatia* dispersed in 1 M of acetate buffer with a pH of 5.0 (glucuronidase and sulfatase activity: 300 and 3 U/mL of buffer, respectively); the process was stopped the next day by the addition of 750 µL of formic acid. The deconjugation procedure described above is an adapted protocol used elsewhere [61]. Following mixing and centrifugation, the supernatants were loaded on Bond Elut Plexa 30 mg cartridges preconditioned sequentially with 1 mL of ethyl acetate, 1 mL of 1% formic acid in methanol (*v*/*v*), and 1 mL of 1% formic acid in water (*v*/*v*). After loading, the cartridges were washed with 1 mL of 1% formic acid (*v*/*v*) in 15% methanol (*v*/*v*) and dried for 30 min in an SPE dryer connected to a vacuum pump. Analytes were eluted with 4 × 250 µL of ethyl acetate, similarly to the work of Klimowska et al., 2023 [62], and then carefully evaporated just to dryness at 40 °C under a nitrogen stream. The reconstitution comprised several steps. Firstly, 40 µL of methanol and 10 µL of water were added to the tube. After the addition of each of the solvents, the content was vortexed at 2000 rpm. Then, to prevent the loss of the extract, the tubes were centrifuged for 2 min at 1500 rpm. Finally, the entire extract was transferred to centrifugal filters (nylon, pore size 0.2 µm) and centrifuged for 3 min at 14,000× *g*. The content of centrifugal tubes was then transferred to amber glass vials containing glass microinserts and injected into an LC-MS/MS system.

**Figure 5:**
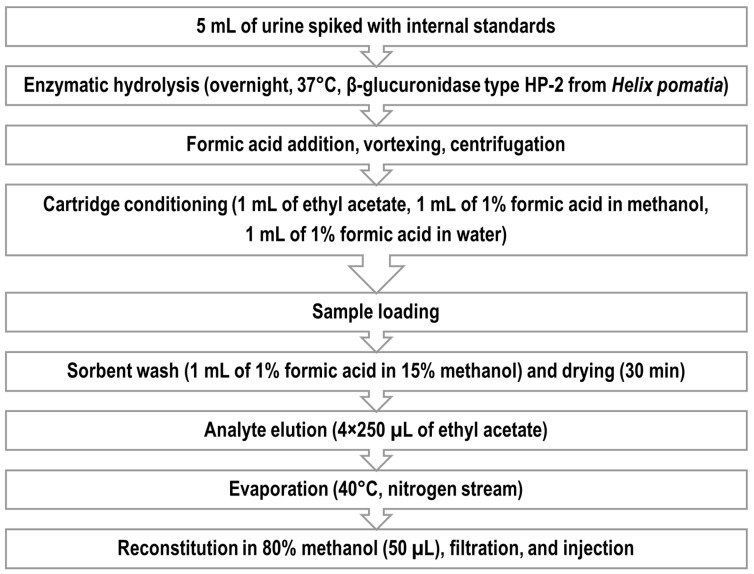
Workflow of the final sample preparation procedure for urine samples.

The outline of the procedure is shown below (Figure 5); a description of optimization experiments follows.

### 3.5. Method Development

#### 3.5.1. Filtration Loss Experiment

The following solvent compositions were tested: 60, 80, and 100% methanol (*v*/*v*). To cover a wide range of lipophilicity, mixture of three compounds in acetonitrile was prepared: IMI (80,000 pg/mL; logP 0.6), FIP (2000 pg/mL; logP 3.8), and *cis*-PER (160,000 pg/mL; logP 6.1) [68]. A sample of 100 µL of the mixture was added to glass tubes and evaporated. The reconstitution was performed as follows: first, methanol was added and the tube content was mixed, then water was transferred into the tube followed by short vortexing. For each methanol percentage, three replicates were prepared and filtered. Since the solvent composition of a sample injected into an LC system is known to affect peak shape and height [65], three unfiltered samples with the same methanol content acted as a reference for every tested percentage (100% recovery).

#### 3.5.2. Extraction Cartridge Selection

Pooled urine used for this experiment was split in half; one was left unchanged, whereas the other was spiked before extraction with a mixture of standards so that the final analyte concentrations in urine were as follows:

- 20 pg/mL: FIP-desulfinyl, FIP, FIP-sulfide, FIP-sulfone;
- 100 pg/mL: DEET, FIP-amide, PFOA;
- 500 pg/mL: BPS, DPhP, FIP-dtfms, TEB-OH;
- 1000 pg/mL: 4F3PBA;
- 2000 pg/mL: IMI;
- 5000 pg/mL: IMZ-OH, BOS-OH;
- 10,000 pg/mL: IMI-OH, 4OH3PBA, CPhCA.

Three aliquots of spiked and unspiked urine were loaded on each type of the cartridges that were preconditioned with 1 mL (Bond Elut Plexa 30 mg) or 2 mL (Oasis HLB 60 mg) of 1% formic acid in methanol (*v*/*v*) and 1% formic acid in water (*v*/*v*). Additionally, a single reagent and urine blank for both cartridges was prepared, so eight samples in total were run per sorbent. The washing step was performed using 1% formic acid (*v*/*v*) in 5% methanol (*v*/*v*); again, 1 mL of washing solution was added to Plexa, and 2 mL was added to Oasis cartridges. After the drying step, the analytes were eluted using 1 mL (Plexa) or 2 mL (Oasis) of ethyl acetate; at that moment, the mixture of analytes corresponding to 100% recovery was added to three unspiked urine extracts per sorbent. After the evaporation of ethyl acetate under a nitrogen stream, the dry residue was reconstituted using 80% methanol (*v*/*v*) and injected into the LC-MS/MS system.

#### 3.5.3. Washing Step Optimization

This experiment was conducted using Bond Elut Plexa in 30 mg only. Samples of 1% formic acid (*v*/*v*) in 0, 5, 10, 15, 20, and 25% methanol (*v*/*v*) were tested (n = 3). The same (un)spiked urine as in the previous section was used, and the rest of the SPE procedure remained unchanged.

### 3.6. Method Validation

Method validation was performed basing on EMA ICH M10 guidelines [69], with the exception of matrix effect investigation, which was performed in accordance with earlier EMA guidelines [70]. A detailed description of performed experiments is provided below.

#### 3.6.1. Selectivity

Selectivity as a capability of an analytical method to differentiate and measure the analyte(s) despite the presence of interferences was assessed by injecting several blank samples from separate sources. The acceptable threshold was less than or equal to 20% of the analyte response at the LLOQ level and no more than 5% of the IS response in the LLOQ sample.

#### 3.6.2. Internal Standard Selection and Matrix Effect

To investigate the matrix effect, seven different lots of urine were prepared in triplicate (SG range 1.006–1.031, determined refractometrically) and fortified post-extraction with analytes. Internal standards were added as well, in amounts corresponding to the following concentrations in urine (pg/mL): IMI-D_4_, 10,000; BPS-D_8_, 5000; FIP-dtfms-^13^C_2_^15^N_2_, 25,000; 3PBA-^13^C_4_, 50,000; FIP-^13^C_4_, 1000. In parallel, three repetitions of analyte and IS mixture in pure solvent were prepared. Following analysis, the matrix factor was calculated as the ratio of analyte peak area in the blank matrix to the analyte peak area in pure solvent and was expressed in %. A result equal to 100% indicates no matrix effect, while results below that indicate signal suppression, and values above 100% reflect signal enhancement. The IS-normalized matrix factor was measured by dividing the matrix factor of a given analyte by the matrix factor of the IS. A CV not greater than 15% for an IS-normalized matrix factor was considered satisfactory. For each analyte, all ISs available were tested; the one that provided the lowest CV of the IS-normalized matrix factor was chosen for routine analysis.

#### 3.6.3. Linearity and Lower Limit of Quantification

The calibration curves were prepared by spiking a blank urine matrix at 10 calibration levels for all analytes. Blank and zero samples (blank sample spiked with IS) were run in parallel. The calibration range was based on pre-validation studies on assay sensitivity and expected concentrations in real samples. The curves were prepared and run in quadruplicate over a four-day period. For LLOQ determination, the acceptable accuracy of each standard was ±20% of the nominal concentration; for other levels, accuracy within ±15% was considered sufficient. A minimum of 75% of the samples at each calibration level had to meet the aforementioned criteria. For each analyte, curve fitting and weighting were assessed using the MS Workstation software, version 6.9.3. The linearity was monitored using R^2^; a value above 0.9900 was considered acceptable.

#### 3.6.4. Accuracy and Precision

The QC samples were prepared from a single source of a blank matrix at two concentration levels: low (LQC) and high (HQC). Within-run accuracy and precision were determined by analyzing 5 replicates at both concentration levels in a single run; 15 replicates over three days were run and combined to assess between-run accuracy and precision. In both experiments, accuracy within ±15% of the nominal concentration and precision (measured as CV) less than or equal to 15% were considered acceptable.

In non-validation runs, usually consisting of 48 samples, two LQC and HQC samples were run. At least three had to be within ±15% of the nominal values for the run to be accepted.

#### 3.6.5. Carry-Over

Carry-over was assessed by the analysis of blank solvent analysis preceded by the injection of a sample at the highest calibration level. The maximum acceptable carry-over was 20% of the LLOQ for the analytes and 5% of the response for the internal standards.

#### 3.6.6. Dilution Integrity

The same urine lot as was used for the accuracy and precision experiments was spiked with analytes at a level eight times higher than the ULOQ and prepared normally in five replicates. Before instrumental analysis, the samples were diluted eightfold with blank solvent. Accuracy and precision were determined and acceptance criteria were the same as for LQC and HQC samples (see above).

#### 3.6.7. Stability

Several stability studies were conducted. In all cases, the samples were run in triplicates. The accuracy needed to be within 15% of nominal concentration and a precision (CV) less than or equal to 15% for the result to be accepted. Firstly, a 24 h autosampler stability of processed samples at room temperature was performed. The LQC and HQC urine samples prepared according to the final protocol (see Section 3.4.) were injected at t = 0 and t = 24 h. Secondly, a single batch of unprocessed LQC and HQC samples was used to determine the 30-day and 12-month storage stability at −20 °C. Before the 12-month stability study was conducted, the stability of the standard mixture used to prepare calibration curves at the same storage conditions was investigated.

### 3.7. Method Application

Following validation, the method was used to quantitate the compounds of interest in 28 human urine samples collected in 2021–2022 from Polish pet owners who recently applied an ectoparasiticidal medication on their pet. As pet owners are expected to be dermally exposed to veterinary drugs by direct contact with their pet(s) following ectoparasiticide treatment [80,100], this population seemed particularly fit for the method application study performed here. The detailed methodology and results of that study will be a subject of a separate publication. Apart from volumetric concentrations, the SG-corrected results were also calculated to take the fluctuations in urine dilution into account [101]. Since each of the participants provided several samples, the data cannot be considered as independent; therefore, no correlation analysis was performed.

## Figures and Tables

**Table 1:** Matrix factors (%) for analytes included in the method for urinalysis.

Analyte	Nominal Concentration (pg/mL)	Specific Gravity of Urine Lots							Mean
		1.006	1.010	1.011	1.017	1.026	1.028	1.031	
		Matrix Factors (%)							
IMI-OH	40,000	50	52	51	50	10	44	49	44
IMI	8000	43	32	40	40	21	37	51	38
BPS	2000	17	12	13	11	15	10	16	13
BOS-OH	20,000	5	3	2	2	1	1	3	2
4OH3PBA	40,000	16	6	8	7	3	4	4	7
DPhP	2000	89	72	61	64	25	34	42	55
DEET	400	38	20	24	23	22	21	18	24
FIP-dtfms	2000	38	19	18	15	9	10	7	17
FIP-amide	400	19	11	14	10	5	6	5	10
TEB-OH	2000	81	58	56	48	42	45	34	52
4F3PBA	4000	14	10	11	12	6	9	10	10
FIP-desulfinyl	80	67	40	34	36	30	32	21	37
FIP	80	71	48	52	48	34	39	26	46
FIP-sulfide	80	53	37	37	36	5	27	12	30
FIP-sulfone	80	61	31	41	35	28	28	20	35
CPhCA	40,000	55	31	39	33	28	29	27	35

**Table 2:** IS-corrected matrix factors (%) and their respective CVs for analytes quantitated in urine. The analyte concentrations are provided in Table 1.

Analyte	Internal Standard	Specific Gravity of Urine Lots							Mean	CV
		1.006	1.010	1.011	1.017	1.026	1.028	1.031		
		IS-Corrected Matrix Factors (%)								
IMI-OH	IMI-D_4_	110	146	114	117	38	106	85	102	30
IMI	IMI-D_4_	93	91	90	95	78	88	90	89	6
BPS	BPS-D_8_	85	74	74	60	79	61	74	72	12
BOS-OH	BPS-D_8_	24	16	13	11	6	8	14	13	41
4OH3PBA	FIP-dtfms-^13^C_2_^15^N_2_	48	36	44	47	42	44	60	46	15
DPhP	FIP-^13^C_4_	118	140	106	125	69	80	150	112	25
DEET	FIP-^13^C_4_	51	38	42	46	60	48	64	50	18
FIP-dtfms	FIP-dtfms-^13^C_2_^15^N_2_	114	110	105	110	111	106	116	110	3
FIP-amide	FIP-dtfms-^13^C_2_^15^N_2_	57	62	80	72	65	69	78	69	11
TEB-OH	FIP-^13^C_4_	108	112	96	93	113	104	123	107	9
4F3PBA	3PBA-^13^C_6_	52	79	65	71	74	66	89	71	15
FIP-desulfinyl	FIP-^13^C_4_	89	78	58	70	82	73	76	75	12
FIP	FIP-^13^C_4_	95	93	90	95	93	91	95	93	2
FIP-sulfide	FIP-^13^C_4_	71	72	64	71	15	62	41	57	35
FIP-sulfone	FIP-^13^C_4_	80	60	70	68	76	66	72	70	9
CPhCA	FIP-^13^C_4_	73	60	68	65	76	68	96	72	15

**Table 3:** Internal standard selection, linearity data and sensitivity obtained for analytes during method validation.

Analyte	IS	LLOQ (pg/mL)	Linear Range ^1^ (pg/mL)	Regression Equation	Curve Fit	Curve Weighting	Coefficient of Determination (R^2^)
IMI-OH	IMI-D_4_	1000	1000–200,000	0.0853x + 0.0065	Linear	1/x	0.9972
IMI	IMI-D_4_	100	100–20,000 (160,000)	1.5555x + 0.0017	Linear	1/x	0.9990
BPS	BPS-D_8_	50	50–5000 (40,000)	0.2526x + 0.0056	Linear	1/x	0.9979
BOS-OH	BPS-D_8_	1000	1000–200,000	0.0138x + 0.0020	Linear	1/x	0.9969
4OH3PBA	FIP-dtfms-^13^C_2_^15^N_2_	2000	2000–200,000	0.0185x − 0.0018	Linear	1/x	0.9907
DPhP	FIP-^13^C_4_	100	100–5000	0.0668x + 0.0238	Linear	1/x	0.9935
DEET	FIP-^13^C_4_	10	10–2000	0.8049x + 0.0604	Linear	1/x	0.9991
FIP-dtfms	FIP-dtfms-^13^C_2_^15^N_2_	200	200–40,000 (320,000)	0.8586x − 0.0021	Linear	1/x	0.9995
FIP-amide	FIP-dtfms-^13^C_2_^15^N_2_	50	50–5000	0.2650x − 0.0004	Linear	1/x	0.9948
TEB-OH	FIP-^13^C_4_	100	100–20,000	0.0499x + 0.0096	Linear	1/x	0.9973
4F3PBA	3PBA-^13^C_6_	100	100–10,000	3.8030x + 0.0050	Linear	1/x^2^	0.9921
FIP-desulfinyl	FIP-^13^C_4_	5	5–1000 (8000)	0.9155x + 0.0027	Linear	1/x	0.9988
FIP	FIP-^13^C_4_	1	1–200 (1600)	1.2291x + 0.0013	Linear	1/x	0.9996
FIP-sulfide	FIP-^13^C_4_	1	1–200 (1600)	0.8579x + 0.0007	Linear	1/x	0.9992
FIP-sulfone	FIP-^13^C_4_	0.5	0.5–100 (800)	1.3612x + 0.0053	Linear	1/x	0.9995
CPhCA	FIP-^13^C_4_	500	500–100,000 (800,000)	0.0012x + 0.0015	Linear	1/x	0.9993

^1^ The values in brackets are quantifiable after eightfold dilution (see Section 2.3.6.).

**Table 4:** Accuracy and precision investigation for analytes in urine at LQC and HQC level.

Analyte	LQC					HQC				
	Nominal Concentration (pg/mL)	Accuracy (%)		Precision (CV, %)		Nominal Concentration (pg/mL)	Accuracy (%)		Precision (CV, %)	
		Intra-Run (n = 5)	Inter-Run (n = 15)	Intra-Run (n = 5)	Inter-Run (n = 15)		Intra-Run (n = 5)	Inter-Run (n = 15)	Intra-Run (n = 5)	Inter-Run (n = 15)
IMI-OH	3000	102	86	13	20	50,000	96	95	8	8
IMI	300	99	100	6	8	5000	97	101	4	9
BPS	75	97	89	14	14	1250	110	102	7	10
BOS-OH	3000	100	100	9	13	50,000	101	96	7	12
4OH3PBA	3000	101	97	12	14	50,000	88	86	5	7
DPhP	150	91	101	8	16	2500	100	86	7	14
DEET	30	105	113	15	14	500	97	104	3	4
FIP-dtfms	600	100	98	9	6	10,000	102	102	3	3
FIP-amide	150	95	88	7	13	2500	96	95	7	6
TEB-OH	300	104	100	8	10	5000	103	103	4	6
4F3PBA	150	95	93	11	13	2500	97	99	4	6
FIP-desulfinyl	15	99	97	6	8	250	100	103	4	4
FIP	3	97	93	7	12	50	99	97	4	7
FIP-sulfide	3	102	96	10	14	50	95	99	7	11
FIP-sulfone	1.5	103	88	10	13	25	97	100	7	14
CPhCA	1500	93	99	10	12	25,000	99	101	3	4

**Table 5:** Results of the stability study for the compounds of interest. The 24 h results refer to the stability of processed samples at room temperature, whereas the 30-day and 12-month values describe the stability of unprocessed samples stored at −20 °C.

Analyte	Study Period	LQC			HQC		
		Nominal Concentration (pg/mL)	Accuracy (n = 4, %)	Precision (n = 4, CV, %)	Nominal Concentration (pg/mL)	Accuracy (n = 4, %)	Precision (n = 4, CV, %)
	24 h		88	13		96	8
IMI-OH	30-day	3000	86	15	50,000	95	6
	12-month		92	12		90	4
	24 h		99	7		103	9
IMI	30-day	300	99	14	5000	100	5
	12-month		108	14		103	3
	24 h		90	10		103	7
BPS	30-day	75	115	4	1250	104	9
	12-month		113	14		108	6
	24 h		101	6		85	3
BOS-OH	30-day	3000	80	8	50,000	89	1
	12-month		46	11		46	11
	24 h		88	7		89	4
4OH3PBA	30-day	3000	107	4	50,000	79	7
	12-month		77	10		58	2
	24 h		105	8		86	4
DPhP	30-day	150	109	13	2500	87	9
	12-month		97	14		114	10
	24 h		113	5		110	2
DEET	30-day	30	115	6	500	108	6
	12-month		97	6		98	5
	24 h		94	11		105	2
FIP-dtfms	30-day	600	97	1	10,000	105	3
	12-month		98	1		99	2
	24 h		89	6		91	6
FIP-amide	30-day	150	105	11	2500	93	6
	12-month		102	8		99	3
	24 h		96	11		100	2
TEB-OH	30-day	300	97	6	5000	101	10
	12-month		102	10		99	2
	24 h		91	15		100	6
4F3PBA	30-day	150	99	14	2500	91	6
	12-month		115	13		92	4
	24 h		94	11		106	4
FIP-desulfinyl	30-day	15	93	3	250	102	2
	12-month		98	14		90	3
	24 h		90	5		90	1
FIP	30-day	3	89	1	50	90	4
	12-month		96	0		89	1
	24 h		91	13		92	0
FIP-sulfide	30-day	3	90	10	50	92	4
	12-month		95	12		85	5
	24 h		89	12		89	4
FIP-sulfone	30-day	1.5	86	9	25	89	5
	12-month		85	3		86	10
	24 h		97	2		105	1
CPhCA	30-day	1500	95	3	25,000	99	4
	12-month		96	12		108	2

**Table 6:** Results of the applicability study (n = 28).

Analyte	% ≥LLOQ ^1^	Unadjusted (pg/mL)						Specific Gravity-Adjusted (pg/mL)					
		GM	P25	P50	P75	P95	Max	GM	P25	P50	P75	P95	Max
IMI-OH	0	-	-	-	-	-	-	-	-	-	-	-	-
IMI	4	-	-	-	-	-	101	-	-	-	-	-	122
BPS	89	412	277	663	791	2888	3947	484	257	537	840	2437	2978
BOS-OH	50	1238	-	538 ^2^	2732	11,950	14,190	1454	-	561	3726	13,374	13,384
4OH3PBA	18	-	-	-	-	9813	14,008	-	-	-	-	8811	13,350
DPhP	93	1541	952	1712	3531	>ULOQ ^3^	>ULOQ	1810	930	1656	3692	>ULOQ	>ULOQ
DEET	68	20.2	-	15.4	78.5	212	214	23.7	-	17.7	101	190	216
FIP-dtfms	0	-	-	-	-	-	-	-	-	-	-	-	-
FIP-amide	0	-	-	-	-	-	-	-	-	-	-	-	-
TEB-OH	86	281	134	259	741	4565	6905	330	108	254	580	3012	4465
4F3PBA	4	-	-	-	-	-	416	-	-	-	-	-	290
FIP-desulfinyl	0	-	-	-	-	-	-	-	-	-	-	-	-
FIP	71	3.94	-	3.32	9.98	129	150	4.63	-	3.39	24.9	233	306
FIP-sulfide	0	-	-	-	-	-	-	-	-	-	-	-	-
FIP-sulfone	39	-	-	-	0.858	16.7	17.7	-	-	-	1.04	14.5	16.9
CPhCA	0	-	-	-	-	-	-	-	-	-	-	-	-

^1^ LLOQ, GM, P25-95 and Max stand for lower limit of quantification, geometric mean, 25–95th percentile, and maximum, respectively. ^2^ Since the detection rate of BOS-OH was exactly 50%, this value is an arithmetic mean of zero and the first non-zero result. ^3^ ULOQ, upper limit of quantification.

**Table 7:** Compound-specific parameters of analytes and internal standards used for LC-MS/MS analysis.

Compound	Status	Retention Time (min)	Precursor Ion	Precursor *m*/*z*	Capillary Voltage (V)	Product Ions *m*/*z* ^1^	Collision Energy (V) ^2^
IMI-OH	analyte	4.90	[M+H]^+^ [M−H]^−^	272.0 270.0	70 −70	225.0, 228.0 46.1	14, 10 10
IMZ-OH	analyte	5.18	[M+H]^+^	257.0	90	69.0, 125.0, 136.0	19, 33, 42
IMI	analyte	5.33	[M+H]^+^	256.0	70	209.0, 175.2, 212.2	12, 14, 11
BPS	analyte	6.11	[M−H]^−^	249.1	−110	155.4, 91.9, 107.9	21, 33, 27
BOS-OH	analyte	8.31	[M−H]^−^ [M+H]^+^	357.0 359.0	−100 100	243.7 140.0, 323.0	18 21, 20
4OH3PBA	analyte	8.51	[M−H]^−^	229.3	−70	108.9, 109.8, 185.6	21, 20.5, 12
DPhP	analyte	8.52	[M−H]^−^	249.0	−92	92.7, 154.6	26, 19.5
DEET	analyte	9.09	[M+H]^+^	192.0	88	119.0, 91.0	14, 26
FIP-dtfms	analyte	9.14	[M−H]^−^	319.0	−70	282.9, 262.8	8, 20
FIP-amide	analyte	9.26	[M−H]^−^	453.0	−70	347.8, 271.9, 303.8	15, 41, 25
TEB-OH	analyte	10.04	[M+H]^+^	324.0	90	70.0, 125.0, 179.1	20, 37, 19
4F3PBA	analyte	10.39	[M−H]^−^	230.9	−75	92.7, 186.6	25, 10
FIP-desulfinyl	analyte	10.77	[M−H]^−^	387.0	−50	350.9, 281.9, 330.8	12, 30, 28
FIP	analyte	10.96	[M−H]^−^	435.0	−70	329.7, 249.6, 277.6	15, 26, 27
FIP-sulfide	analyte	11.12	[M−H]^−^	419.0	−70	261.8, 313.9, 382.9	26, 18, 11
FIP-sulfone	analyte	11.34	[M−H]^−^	451.0	−70	281.9, 243.8, 414.9	25, 44, 17
PFOA	analyte	11.79	[M−H]^−^ [M−CO_2_−H]^−^	413.0 369.0	−30 −72	369.0, 169.0 219.0	7, 18.5 10.5
CPhCA	analyte	12.11	[M−H]^−^	283.0	−90	246.6, 35.2	7, 9
IMI-D_4_	internal standard	5.30	[M+H]^+^	260.0	70	213.0, 214.0, 216.0	12, 8, 10
BPS-D_8_	internal standard	6.05	[M−H]^−^	257.0	−110	112.0, 96.0, 160.0	27, 33, 21
FIP-dtfms-^13^C_2_^15^N_2_	internal standard	9.13	[M−H]^−^	323.0	−70	287.0, 184.9	8, 28
3PBA-^13^C_4_	internal standard	10.40	[M−H]^−^	219.0	−90	99.0, 175.0	19, 11
FIP-^13^C_4_	internal standard	10.97	[M−H]^−^	439.0	−70	334.0, 250.9, 321.9	15, 26, 24

^1^ First ion is the quantifier, the others are the qualifiers (in increasing *m*/*z* order). ^2^ For product ions, respectively.

## Data Availability

The raw data supporting the conclusions of this article will be made available by the authors on request.

## Supplementary Materials

The following supporting information can be downloaded at https://www.mdpi.com/article/10.3390/ijms26189025/s1.

## Abbreviations

The following abbreviations are used in this manuscript:

HBM	Human biomonitoring
PFAS	Per-/polyfluoroalkyl substance
FIP	Fipronil
IMI	Imidacloprid
EU	European Union
FIP-sulfone	Fipronil-sulfone
FIP-hydroxy	Fipronil-hydroxy
FIP-dtfms	Fipronil-detrifluoromethylsulfinyl
FIP-amide	Fipronil-amide
FIP-desulfinyl	Fipronil-desulfinyl
FIP-sulfide	Fipronil-sulfide
FIPs	Fiproles
IMI-OH	Imidacloprid-5-hydroxy
4OH3PBA	4′-hydroxy-3-phenoxybenzoic acid
CPhCA	3-(2-chloro-2-(4-chlorophenyl)vinyl)-2,2-dimethylcyclopropanecarboxylic acid
4F3PBA	4-fluoro-3-phenoxybenzoic acid
IMZ-OH	Imazalil-despropenyl
BOS-OH	Boscalid-5-hydroxy
TEB-OH	Tebuconazole-*tert*-butylhydroxy
DEET	*N*,*N*-Diethyl-*meta*-toluamide
BPS	Bisphenol S
OPFRs	Organophosphate flame retardants
DPhP	Diphenyl phosphate
PFOA	Perfluorooctanoic acid
LC-MS/MS	Liquid chromatography-tandem mass spectrometry
SPE	Solid phase extraction
*cis*-PER	*cis*-Permethrin
SG	Specific gravity
IS	Internal standard
CV	Coefficient of variation
LLOQ	Lower limit of quantification
R^2^	Coefficient of determination
LOQ	Limit of quantification
LOD	Limit of detection
ULOQ	Upper limit of quantification
EMA	European Medicines Agency
QC	Quality control
LQC	Quality control sample at low concentration
HQC	Quality control sample at high concentration
GM	Geometric mean
