# Supplementary material for: A Sensitive SPE-LC-MS/MS Method for Determination of Selected Veterinary Drugs and Other Organic Contaminants in Human Urine: Development, Validation, and Application Study
Source: Int J Mol Sci. 2025 Sep 16;26(18):9025. doi: 10.3390/ijms26189025 (PMC12469592; doi:10.3390/ijms26189025)
Supplement: Supplementary file 1 [file ijms-26-09025-s001.zip › ijms-3870215-supplementary.pdf]

Supplementary material for

A sensitive SPE-LC-MS/MS method for determination of selected veterinary drugs and other organic contaminants in human urine: development, validation, and application study

Wojciech Rodzaj [1], Małgorzata Wacławik [1], Joanna Jurewicz [2], and Bartosz Wielgomas [1],*

[1] Department of Toxicology, Faculty of Pharmacy, Medical University of Gdańsk, 107 Hallera Street, 80-416 Gdańsk, Poland; wojciech.rodzaj@gumed.edu.pl (W.R.); malgorzata.waclawik@gumed.edu.pl (M.W.); bartosz.wielgomas@gumed.edu.pl (B.W.)

[2] Department of Chemical Safety, Nofer Institute of Occupational Medicine, 8 Teresy Street, 91-348 Łódź, Poland; joanna.jurewicz@imp.lodz.pl (J.J.)

* Correspondence: bartosz.wielgomas@gumed.edu.pl (B.W.)

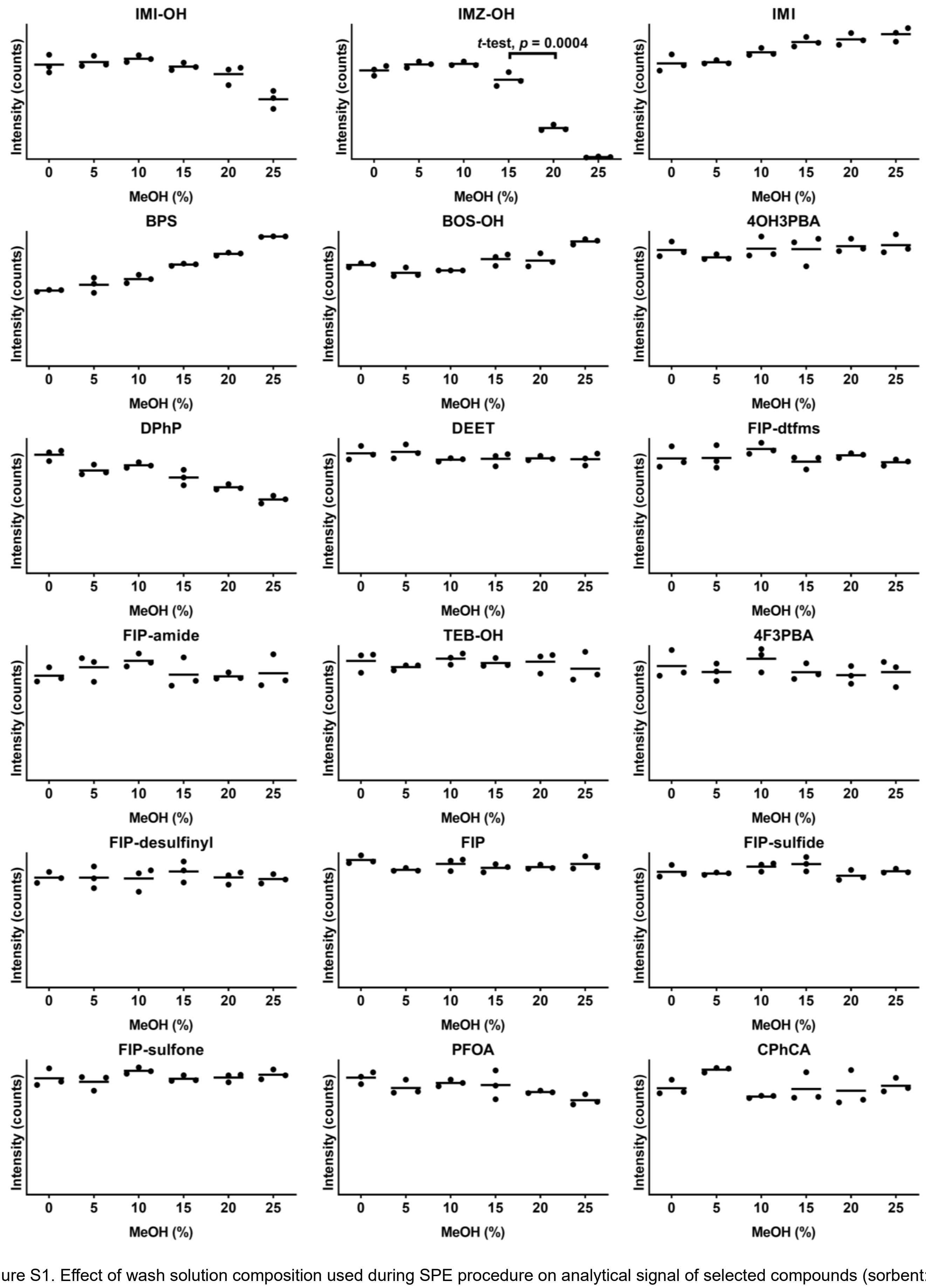

Figure S1. Effect of wash solution composition used during SPE procedure on analytical signal of selected compounds (sorbent: Bond Elut Plexa 30 mg). The horizontal lines represent the average value.

Table S1. Dilution integrity for selected analytes quantified in urine (eightfold dilution, n = 5). For analytes that are not shown in this Table, the results were beyond the accepted range.

Analyte	Accuracy (%)	Precision (CV, %)
IMI	101	6
BPS	85	4
FIP-dtfms	99	4
FIP-desulfinyl	100	5
FIP	104	4
FIP-sulfide	114	4
FIP-sulfone	93	4
CPhCA	93	5

Table S2. Analytical standards used in this work.

Compound	Abbreviation	Status	Group	CAS number	Supplier	Catalog number	Selected parent compound(s)
Imidacloprid-5-hydroxy	IMI-OH	analyte	Pesticides and related compounds	155802-61-2	Witega	PS201	Imidacloprid
Imazalil-despropenyl	IMZ-OH	analyte	Pesticides and related compounds	24155-42-8	Sigma-Aldrich	Y0000137	Imazalil
Imidacloprid	IMI	analyte	Pesticides and related compounds	138261-41-3	Instytut Przemysłu Organicznego	IPO 297	-
Bisphenol S	BPS	analyte	Other	80-09-1	Sigma-Aldrich	43034	-
Boscalid-5-hydroxy	BOS-OH	analyte	Pesticides and related compounds	661463-87-2	Sigma-Aldrich	28001	Boscalid
4'-Hydroxy-3-phenoxybenzoic acid	4OH3PBA	analyte	Pesticides and related compounds	35065-12-4	Roussel Uclaf	RU46606	Permethrin, cypermethrin
Diphenyl phosphate	DPhP	analyte	Organophosphate flame retardants	838-85-7	Aldrich	850608	Triphenyl phosphate
Fipronil-hydroxy	FIP-hydroxy	analyte	Pesticides and related compounds	2304825-80-5	UC Davis	NA	Fipronil
N,*N*-Diethyl-*meta*-toluamide	DEET	analyte	Pesticides and related compounds	134-62-3	Instytut Przemysłu Organicznego	IPO 922	-
Fipronil-detrifluoromethylsulfinyl	FIP-dtfms	analyte	Pesticides and related compounds	120068-79-3	TRC Canada	F342220	Fipronil
Fipronil-amide	FIP-amide	analyte	Pesticides and related compounds	205650-69-7	TRC Canada	D436240	Fipronil
Tebuconazole-*tert*-butylhydroxy	TEB-OH	analyte	Pesticides and related compounds	212267-64-6	Sigma-Aldrich	72843	Tebuconazole
4-Fluoro-3-phenoxybenzoic acid	4F3PBA	analyte	Pesticides and related compounds	77279-89-1	abcr	AB531857	Flumethrin, cyfluthrin
Fipronil-desulfinyl	FIP-desulfinyl	analyte	Pesticides and related compounds	205650-65-3	Sigma-Aldrich	41865	Fipronil
Fipronil	FIP	analyte	Pesticides and related compounds	120068-37-3	Sigma-Aldrich	46451	-
Fipronil-sulfide	FIP-sulfide	analyte	Pesticides and related compounds	120067-83-6	Sigma-Aldrich	34520	Fipronil
Fipronil-sulfone	FIP-sulfone	analyte	Pesticides and related compounds	120068-36-2	Sigma-Aldrich	32333	Fipronil
Perfluorooctanoic acid	PFOA	analyte	Other	335-67-1	Aldrich	171468	-
3-(2-Chloro-2-(4-chlorophenyl)vinyl)-2,2-dimethylcyclopropanecarboxylic acid	CPhCA	analyte	Pesticides and related compounds	88419-72-1	abcr	AB438398	Flumethrin
Imidacloprid-D_4	IMI-D_4	internal standard	Pesticides and related compounds	1015855-75-0	TRC Canada	I274992	-
Bisphenol S-D_8	BPS-D_8	internal standard	Other	NA	TRC Canada	B447392	-
Fipronil-detrifluoromethylsulfinyl-$^{13}C_2{}^{15}N_2$	FIP-dtfms-$^{13}C_2{}^{15}N_2$	internal standard	Pesticides and related compounds	NA	TRC Canada	F342222	-
3-Phenoxybenzoic acid-$^{13}C_6$	3PBA-$^{13}C_4$	internal standard	Pesticides and related compounds	NA	Cambridge Isotope Laboratories	CLM-4542	-
Fipronil-$^{13}C_4$	FIP-$^{13}C_4$	internal standard	Pesticides and related compounds	NA	Sigma-Aldrich	79157	-

Table S3. The parameters of liquid chromatography system used for analysis.

LC part	Parameter name	Parameter value	
Pumping system	Mobile phase A composition	0.5 mM ammonium formate buffer pH 3 in water:methanol 9:1 (*v*/*v*)	
	Mobile phase B composition	0.5 mM ammonium formate buffer pH 3 in methanol	
	Flow rate (mL/min)	0.4	
	Mixer volume (µL)	150	
	Gradient program	Time (min:sec)	%B
		0:00	5
		12:00	100
		17:00	100
		17:01	5
		20:00	5
Autosampler	Temperature	Ambient	
	Injection volume (µL)	10	
	Needle wash solvent composition	Water:methanol:acetonitrile:isopropanol 1:1:1:1 (*v*/*v*/*v*/*v*)	
Column compartment	Temperature (°C)	40	
	Column	ACE Excel 3 SuperC18, 3 µm, 75×3.0 mm	

Table S4. Settings of mass spectrometer used for quantitation of analytes in urine.

Mass spectrometer part	Parameter name	Parameter value
Interface	Ionization type	Electrospray
	Needle voltage (V)	5000, -4500[1]
	Spray shield voltage (V)	600, -600[1]
	Nebulizing gas	Nitrogen, air[1]
	Nebulizing gas pressure (psi)	60
	Drying gas	Nitrogen
	Drying gas pressure (psi)	42
	Drying gas temperature (°C)	220
	Housing temperature (°C)	50
Mass analyzer	Type	Triple quadrupole
	Manifold temperature (°C)	40
	Collision gas	Argon
	Collision gas pressure (mTorr)	2.4
	Mass resolution (amu)	0.7
Detector	Detector voltage (V)	1500

[1] For positive and negative ionization, respectively.
